# Genetic Instability Persists in Non-Neoplastic Urothelial Cells from Patients with a History of Urothelial Cell Carcinoma

**DOI:** 10.1371/journal.pone.0086162

**Published:** 2014-01-22

**Authors:** João Paulo de Castro Marcondes, Maria Luiza Cotrim Sartor de Oliveira, Alisson M. Gontijo, João Lauro Viana de Camargo, Daisy Maria Fávero Salvadori

**Affiliations:** 1 UNESP – Univ. Estadual Paulista, Faculdade de Medicina Botucatu SP, Brazil; 2 Centro de Estudos de Doenças Crônicas, Faculdade de Ciências Médicas (FCM), Universidade Nova de Lisboa, Lisboa, Portugal; University Medical Center Hamburg-Eppendorf, Germany

## Abstract

Bladder cancer is one of the most common genitourinary neoplasms in industrialized countries. Multifocality and high recurrence rates are prominent clinical features of this disease and contribute to its high morbidity. Therefore, more sensitive and less invasive techniques could help identify individuals with asymptomatic disease. In this context, we used the micronucleus assay to evaluate whether cytogenetic alterations could be used as biomarkers for monitoring patients with a history of urothelial cell carcinoma (UCC). We determined the frequency of micronucleated urothelial cells (MNC) in exfoliated bladder cells from 105 patients with (n = 52) or without (n = 53) a history of UCC, all of whom tested negative for neoplasia by cytopathological and histopathological analyses. MNC frequencies were increased in patients with a history of UCC (non-smoker and smoker/ex-smoker patients *vs* non-smoker and smoker/ex-smoker controls; *p*<0.001), in non-smoker UCC patients (*vs* non-smoker controls; *p*<0.01), and in smoker/ex-smoker controls (*vs* non-smoker controls; *p*<0.001). Patients with a history of recurrent disease also demonstrated a higher MNC frequency compared to patients with non-recurrent neoplasia. However, logistic regression using smoking habits, age and gender as confounding factors did not confirm MNC frequency as a marker for UCC recurrence. Fluorescent *in situ* hybridization analysis (using a pan-centromeric probe) showed that micronuclei (MN) arose mainly from clastogenic events regardless of UCC and/or smoking histories. In conclusion, our results confirm previous indications that subjects with a history of UCC harbor genetically unstable cells in the bladder urothelium. Furthermore, these results support using the micronucleus assay as an important tool for monitoring patients with a history of UCC and tumor recurrence.

## Introduction

Bladder cancer is one of the most common genitourinary neoplasms in industrialized countries, and cigarette smoking is the main risk factor for this disease, as smokers have an approximate 3-fold increased risk of disease [1]. Urothelial cell carcinomas (UCC) account for 90% of all urinary bladder tumors, and multifocality and high recurrence rates are important clinical features of this disease [2], [3]. Another critical feature of UCC is the high morbidity associated with the periodic surgical procedures required to investigate tumor recurrence and eventual resection [4]. Tumor grade, stage, size, and multifocality may predict progression and recurrence, although in some patients, these variables fail to accurately predict progression and recurrence because tumors of similar grade and stage can still differ significantly in their biology [5]. The development of cytogenetic markers has increased the sensitivities of UCC diagnosis and prognosis, but the precise characterization of atypical urinary cells remains a challenge for pathologists, especially in cases of low-grade tumors [6].

Cancer cells typically harbor chromosomal alterations, such as aneuploidy or polyploidy and abnormal chromosome structures [7]. The micronuclei (MN) frequency is a widely used biomarker for chromosome instability [8]–[13]. MN are chromosome fragments or whole chromosomes that are not incorporated into daughter nuclei during cell division [8], and the frequency of micronucleated cells (MNC) may predict cancer risk, suggesting that increased MN formation may be associated with early carcinogenic events [9]. Indeed, one retrospective study reported an increased MN frequency in urinary cytology smears from samples that were initially diagnosed as “atypical urothelial cells” but later determined to be malignant cells upon follow-up histopathological analysis [10]. These findings strengthen the notion that MN scoring could be used in cytopathological analyses to assist in the proper diagnosis of these cases [10]. We have previously shown that genetic instability could be detected in urothelial cells, even before cellular atypia was detectable by cytology [13]. In this previous study, increased MNC frequencies were observed in normal urothelial cells obtained from bladder washings of patients with a history of UCC and/or smoking, but this increase was not statistically significant when compared to urothelial cells from a control population, which was likely due to the relatively small sample size [13]. This finding, together with recent data from Arora *et al.* [10], prompted us to investigate the utility of a MN assay to detect genetic instability in urothelial cells with normal cytology in a new and larger cohort of patients with recurrent UCC. Finally, to improve our understanding of the genetic mechanisms involved in UCC, we also performed fluorescent *in situ* hybridization (FISH) in urothelial cells using a human pan-centromeric probe to gain insight into the mechanism by which MN originate in these cells.

## Materials and Methods

### 1. Subjects

This study was approved by the Ethics Committee for Human Research of Botucatu Medical School, UNESP (Document No 276/2005-CEP), and signed informed consent was obtained from all subjects recruited for the present study.

The screening for micronucleated urothelial cells (hereafter referred to as MNCs) was conducted on 52 patients (43 males and 9 females) with a history of UCC and a negative cytopathological diagnosis for neoplasia. The reference group (controls) consisted of 53 patients (32 males and 21 females) with no history of UCC who were scheduled for cystoscopy to investigate other urinary tract complaints (e.g., hematuria, dysuria, pollakiuria, nocturia, vesical and kidney stones, cystitis, neurogenic bladder). UCC history was confirmed from previous positive biopsies, cytological analyses, and medical records. Tumors were classified as low or high grade according to the WHO classification [14]. The UCC patient and control groups were aged 38–89 years and 35–81 years, respectively. The studied population included 3 self-identified ethnic groups, as defined by The Brazilian Institute of Geography and Statistics [15]: white (96.4%), yellow (Asians; 1.8%), and brown (mixed race; 1.8%). Both the patient and control groups were randomly recruited at the Clinical Hospital in Botucatu, São Paulo State (22°53′09?S, 48°26′42?W). The city of Botucatu is a *Schistosoma haematobium* non-endemic region. Personal data for all individuals were recorded in a detailed questionnaire that was administered immediately before or after cystoscopy. The collected data included ethnicity, age, gender, smoking and alcohol consumption habits, medical data, and histories of relevant exposures to organic solvents, dyes, diesel exhaust, pesticides, and X-ray radiation. Subjects were considered smokers if they smoked at least 100 cigarettes in their life and currently smoke every day or some days; ex-smokers were subjects who had stopped smoking at least 1 year prior to sample collection [16]. Alcohol consumption was classified in 3 subjective categories [13]: non-drinker, defined as no alcohol consumption or social drinking; mild drinker, defined as the consumption up to 1 cup (∼3.4 fl. oz) of an alcoholic beverage per day or more than 1 cup on weekends; or heavy drinker, defined as the consumption of more than 1 L of a light alcoholic beverage (beer, wine, or cider) or 2 cups of hard liquor (Brazilian cachaça, vodka, or whiskey) per day for at least 6 years. Subjects were considered “exposed to toxic substances” (pesticides, solvents, and diesel exhaust) if they were occupationally exposed or had been exposed for at least 2 years [13].

### 2. Bladder washes

Bladder washes (bladder barbotage) were obtained by intravesical administration of a 0.9% saline solution. Two aliquots of 15 mL were collected per patient; one of the aliquots was subjected to Giemsa staining for MN examination, and the other was used for FISH. For Giemsa staining, bladder washing samples were centrifuged at 1,500 rpm for 10 min, and 200 µL of the obtained cell suspensions were cytocentrifuged at 1,400 rpm for 10 min. For FISH, 200 µL of the cell suspensions were fixed 3 times in a methanol: acetic acid (3∶1) solution. Slides were stored at room temperature for 2 weeks and then at −20°C until staining and analysis.

### 3. Micronucleus test

**Figure 1:**
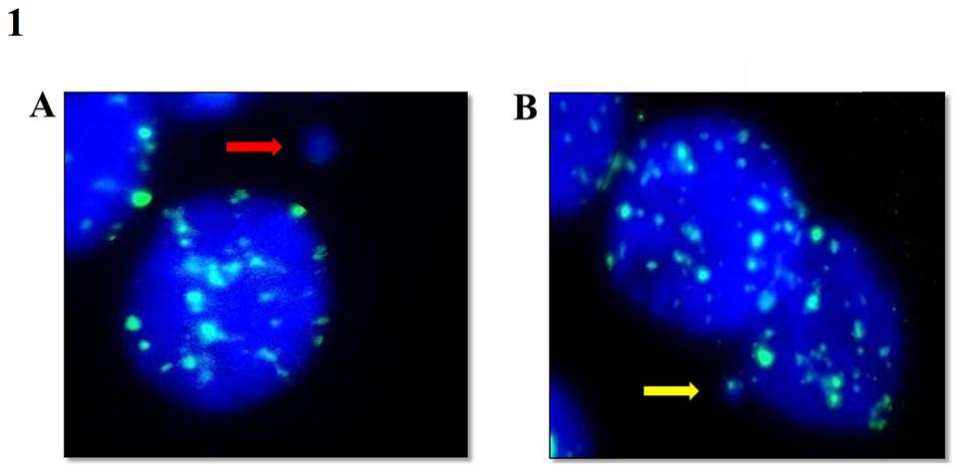
FISH-stained urothelial cells with probes for all chromosome centromeres (green spots). a) Urothelial cells counterstained with DAPI, and the red arrow indicates a micronucleus (MN) with no centromeric signal (MNC−). b) Urothelial cells counterstained with DAPI, and the yellow arrow indicates a MN with centromeric signal (MNC+). Photomicrographs were acquired at 1,000× magnification.

The frequency of MNCs in 5% Giemsa-stained slides was determined by light microscopy at a magnification of 1,000× following the criteria established by Lehucher-Michel *et al.* [12]. Those subjects with frequencies of Giemsa-stained MNCs above 1‰ were selected for FISH MN scoring. The probes used for all human centromeres were directly labeled with FITC (PAHC0001-G, Qbiogene, USA), and FISH slides were processed according to the probe manufacturer's protocol with slight modifications. After the hybridization step, DAPI in antifade solution was used to counterstain DNA (Vector Laboratories, USA), which enabled the simultaneous observation of the total DNA and hybridization signals. MN were examined for fluorescence using a fluorescence microscope at 1,000× magnification with the Case Data Manager software (Applied Spectral Imaging, CA, USA). Cells with non-FITC-labeled MN (centromere-negative MN; MNC−) were assumed to contain acentric chromosome fragments (Figure 1a), whereas FITC-labeled MN (centromere-positive MN; MNC+) contained whole chromosomes (Figure 1b). The frequencies of MNC, MNC+, and MNC− cells per 1,000 cells were calculated for each subject. Due to the large number of degenerate and occasionally scarce urothelial cells, the number of cells analyzed by Giemsa staining ranged from 500 to 1,000 (n = 17, 500–700 cells; n = 21, 701–999; n = 67, 1,000 cells) per subject. For FISH analysis, the number of analyzed cells also ranged from 500 to 1,000 (n = 4, 500–700 cells; n = 2, 701–999; n = 31, 1000 cells).

### 4. Statistical analysis

The number of urothelial cells and MNC were adjusted according to a generalized linear model that considered the MN frequency (MNC, MNC+, and MNC−) as the dependent variable. A binomial distribution was used to adjust the model to consider UCC history, smoking history, and the interaction between UCC and smoking history. The same model was adjusted for gender, age, number of cigarettes per day, duration of smoking, alcohol consumption, and toxic substance exposure. Logistic regression analysis adjusted for demographic variables was used to provide an estimated risk for tumor recurrence in relation to MN frequency. The associations between tumor invasiveness, tumor grade, and tumor recurrence in patients with and without a history of smoking were analyzed using the chi square test. Comparisons of the simple frequencies of gender, tumor invasiveness, tumor grade, tumor recurrence, alcohol consumption, and toxic substance exposure were performed using the test of proportions. All analyses were performed using SAS for Windows (v. 9.2), and a *p* value<0.05 was considered significant.

## Results

The demographic and medical data are presented in Table 1. The number of males (n = 43; 82.7%) with a history of UCC was significantly higher (*p*<0.05) than the corresponding number of females (n = 9; 17.3%). For male and female patients, the average age was 67.8±13.3 years (35–89 years); 31 (59.6%) patients were smokers or ex-smokers, and 21 (40.4%) were non-smokers. No statistically significant differences were observed in the number of non-smoker and smoker/ex-smoker patients. Similarly, there was no statistically significant difference between the number of patients with a history of high-grade (n = 23, 44.2%) or low-grade tumors (n = 29, 55.8%), even when considering smoking habits (*p*>0.05). Regardless of smoking history, the number of subjects diagnosed with invasive tumors (n = 15, 28.8%) was significantly lower compared to the number of subjects with non-invasive tumors (n = 37, 71.2%) (*p*<0.01). Additionally and regardless of smoking history, the number of patients with recurrent tumors was significantly higher (n = 41, 78.8%) than the number of patients with non-recurrent tumors (n = 11, 21.2%) (*p*<0.001). Regarding alcohol consumption and exposure to toxic substances, no significant difference was detected between UCC patients and controls (*p*>0.05). In the control group, 32 (60.4%) patients were male, and 21 (39.6%) female; 29 (57.4%) patients were non-smokers, and 24 (45.3%) were smokers or ex-smokers. The average age was 58.5±13.6 years (35–82 years).

**Table 1:** General characteristics of the study population.

		Control			UCC		
		Non-smokers	Smokers/ex-smokers	Total	Non-smokers	Smokers/ex-smokers	Total
**Number of subjects^1^**		29 (54.7)	24 (45.3)	53	21 (40.4)	31 (59.6)	52
**Gender^2^**	**Male**	55.2	66.7	60.4	71.4	90.3	82.7*
	**Female**	44.8	33.4	39.6	28.6	9.7	17.3
**Age (years)^2^**		59.2±14.2	57.6±13.2	58.5±13.6	67.2±16.4	68.3±10.9	67.8±13.3
**Cigarettes/day^3^**		-	19±10	-	-	26±14	-
**Tumor grade^2^**	**High**	-	-	-	38.1	48.4	44.2
	**Low**	-	-	-	61.9	51.6	55.8
**Tumor invasiveness^2^**	**Invasive**	-	-	-	23.8	32.3	28.8**
	**Non-invasive**	-	-	-	76.2	67.7	71.2
**Tumor recurrence^2^**	**Recurrent**	-	-	-	85.7	74.2	78.8**^#^**
	**Non-recurrent**	-	-	-	14.3	25.8	21.2
**Alcohol consumption ^1;4^**	**Non-drinker**	19 (65.5)	19 (79.2)	38 (71.7)	18 (85.7)	17 (54.8)	35 (67.3)
	**Mild drinker**	2 (6.9)	2 (8.3)	4 (7.5)	1 (4.8)	7 (22.6)	8 (15.4)
	**Heavy drinker**	2 (6.9)	2 (8.3)	4 (7.5)	0 (0)	5 (16.1)	5 (9.6)
**Toxic substance exposure ^1;5^**	**Solvents**	2 (6.9)	8 (33.3)	10 (18.9)	2 (9.5)	8 (25.8)	10 (19.2)
	**Pesticide**	6 (20.7)	7 (29.2)	13 (24.5)	8 (38.1)	6 (19.3)	14 (26.9)
	**Diesel**	1 (3.5)	1 (4.2)	2 (3.8)	1 (4.8)	4 (12.9)	5 (9.6)

UCC, patients with history of urothelial cell carcinoma; ^1^ No. (%); ^2^ data are presented as percentages (%); ^3^ mean ± standard deviation; * *p*<0.05, compared to female patients; ** *p*<0.01, compared to non-invasive tumors; **^#^** *p*<0.001, compared to non-recurrent tumors; ^4^ data not available for 7 controls (6 non-smokers and 1 smoker/ex-smoker) and 4 UCC patients (2 non-smokers and 2 smokers/ex-smokers); ^5^ data not available for 1 smoker/ex-smoker control and 6 UCC patients (2 non-smokers and 4 smokers/ex-smokers).

The MNC frequency (3.0‰) in patients with a history of UCC was significantly higher (*p*<0.001) than the frequency in the control group (1.9‰) (Table 2). However, when considering smoking history, a statistically significant (*p*<0.001) difference was detected only between non-smoker UCC patients (3.7‰) and non-smoker controls (1.3‰). In both the UCC patient and control groups, a statistically significant difference was observed between non-smokers and smokers/ex-smokers (Table 2). Indeed, it is important to notice that there was no statistically significant difference in the MNC frequency of smokers/ex-smokers from the UCC group, when compared with the control group (*p>*0.05). No interference was observed in MNC frequencies when alcohol consumption and toxic substance exposure were included in the binomial analysis.

**Table 2:** Urothelial MNC frequency in subjects with and without a history of bladder UCC.

Groups	Number of subjects	Number of cells analyzed	Number of MNC	‰ MNC^1^	*p* value
**Control**	53	54,804	107	1.9	-
Non-smokers	29	30,639	40	1.3	-
Smokers/ex-smokers	24	24,165	67	2.8^a^	<0.001^a^
**UCC**	52	44,949	135	3.0^b^	<0.001^b^
Non-smokers	21	19,052	70	3.7^c,d^	<0.05^c^, <0.001^d^
Smokers/ex-smokers	31	25,897	65	2.5^e,f^	<0.01^e^, >0.05^f^

1 MNC per 1,000 cells; a, smoker/ex-smoker controls *vs.* non-smoker controls; b, UCC patients (non-smokers + smokers/ex-smokers) *vs.* controls (non-smokers + smokers/ex-smokers); c, non-smoker UCC patients *vs.* smoker/ex-smoker UCC patients; d, non-smoker UCC patients *vs.* non-smoker controls; e, smoker/ex-smoker UCC patients *vs.* non-smoker controls; f, smoker/ex-smoker UCC patients *vs.* smoker/ex-smoker controls.

FISH data were obtained from 37 subjects (21 UCC patients and 16 controls) with greater than 1‰ Giemsa-stained MNC. Although the binomial analyses demonstrated significant differences, data were interpreted qualitatively because of the low number of MNC+ and MNC− samples. Overall, 75% (range, 66.6–81.5%) of the scored MNC samples stained negative for centromeres (MNC−). In fact, the largest increase in MNC− status compared to centromere-positive MNCs (MNC+) was observed for smoker/ex-smoker UCC patients (81.5% MNC− *vs.* 18.5% MNC+) (Table 3).

**Table 3:** Frequencies of centromere-positive (MNC+) and centromere-negative (MNC−) urothelial MNCs from subjects with and without a history of bladder UCC.

Groups	Number of subjects	Total number of			
		MNC	MNC^1^	MNC+ (%)	MNC− (%)
**Control**	16	14,783	20	5 (25)	15 (75)
Non-smokers	8	7,229	4	1 (25)	3 (75)
Smokers/ex-smokers	8	7,554	16*	4 (25)	12* (75)
**UCC**	21	20,150	39	9 (23.1)	30** (76.9)
Non-smokers	8	8,000	12	4 (33.3)	8 (66.6)
Smokers/ex-smokers	13	12,150	27	5 (18.5)	22** (81.5)

1 MNC, micronucleated urothelial cells; *p<0.05, compared to non-smoker controls; **p<0.01, compared to % MNC+ within the UCC group.

When demographic and clinical features were considered (Table 4), significant (*p*<0.05) interference was detected in MNC frequency for age and tumor recurrence status in the UCC patient group. Patients older than 60 years showed higher MNC frequencies compared to patients less than 60 years of age (3.5‰ *vs.* 2.1‰, respectively; *p*<0.05); patients with recurrent tumors also had higher MNC frequencies compared to patients with non-recurrent tumors (3.4‰ *vs.* 1.7‰, respectively; *p*<0.05). Importantly, 70.3% of patients with recurrent tumors (n = 29) were older than 60 years of age. In addition, logistic regression analysis did not reveal a relationship between high MNC frequencies and increased tumor recurrence risk in smokers/ex-smokers UCC patients (*p>*0.05). Age, gender and smoking habit were considered as potential confounding factors in the regression model.

**Table 4:** Urothelial MNC frequency (Giemsa staining) in subjects with and without a history of UCC stratified according to demographic, histological and clinical variables.

Variables		Number of subjects		‰ MNC^1^	
		Control	UCC	Control	UCC
**Gender**	male	32	43	1.8	2.8
	female	21	9	2.2	3.8
**Age**	≤60 years old (35–60 years)	29	16	1.9	2.1
	>60 years old (61–89 years)	24	36**	1.9	3.5*
**Tumor grade**	low grade	-	29	-	2.9
	high grade	-	23	-	3.2
**Tumor recurrence^2^**	non-recurrent	-	11	-	1.7
	recurrent	-	41	-	3.4
**Tumor invasiveness**	non-invasive	-	37	-	3.1
	invasive	-	15	-	2.7

1 MNC – micronucleated cells per 1000 cells; *p<0.05, compared to UCC patients less than 60 years of age; ^2^ Logistic regression analysis (considering smoking habit, age and gender as confounding factors) did not show significant difference, p>0.05.

## Discussion

In this study, we used the micronucleus test to evaluate chromosomal damage in patients with a history of UCC, in an attempt to introduce this test as an auxiliary biomarker for bladder cancer monitoring. We observed a significant increase in MNC frequency in patients with a history of UCC, but with cytological and histopathological diagnoses negative for neoplasia. Our data both corroborate and bring statistical support to the trend of increased MN in UCC, which we previously detected in an independent study with a smaller group of patients with a history of UCC [13]. Furthermore, the present data are highly consistent with increased levels of primary DNA damage and aneuploidy, which we previously detected using the alkaline single-cell gel (comet) assay [13]. Similarly, a recent study reported p53 protein expression in more than 25% of exfoliated urothelial cells from histologically normal urothelium of patients with a history of UCC, which also indicates that this “normal” urothelium is genomically unstable [17]. Additionally, urothelial cells were also analyzed for chromosomal aberrations using high-resolution comparative genomic hybridization and, they did not find numerical alterations of the chromosomes 7, 17 and 9p21 regions, but did find high frequencies of gains in 11p12 and losses in 16p12 [17]. It is important to emphasize that an increased MNC frequency was not predictive of UCC recurrence when a logistic regression was carried out including age, gender and smoking habits as confounding factors. Therefore, taken together our data strongly support the idea that genetic instability can be detected prior to a diagnosis of cellular atypia and highlight MN examination as an important tool for monitoring patients with a history of UCC and recurrent tumors.

To gain insight into the mechanism of MN formation in cytologically normal urothelial cells from patients with a history of UCC, we used FISH with a pan-centromeric probe to evaluate aneugenic and clastogenic events. In the control and UCC patient groups, the frequencies of centromere negative MN (MNC−) were consistently higher than the frequencies of centromere positive MN (MNC+). This result suggested that MN mainly arose from chromosomal, and that this tendency was either stabilized or mildly exacerbated in patients with a history of UCC. In fact, Del Rey *et al.* [18] suggested that MN resulting from acentric fragments (chromosome breakage or cyclin D1 gene amplification) or from whole chromosomes might contribute to genomic instability in UCC tumor samples. Similarly, a high frequency of acentromeric MN was observed in the peripheral lymphocytes of patients with different types of cancer prior to treatment [11].

Our data from control subjects also confirm previous reports in which smoking history was associated with significant increases in MN frequencies [12], [13], [19] and primary DNA lesions [20]. Interestingly, we did not observe the same effect in smoker/ex-smoker UCC patients. Thus, as previously proposed [21], we suggest that the pathological status may mask the effects of cigarette smoking on MNC frequency. Cigarette smoking has also been associated with other genetic alterations found in bladder cancer patients. *TP53* mutations, *TP16* promoter hypermethylation, and the overexpression of various oncogenes and growth factors have been detected in tumor and normal tissues from patients with a history of smoking [22]–[25]. Furthermore, increases in p53 protein levels were observed in the normal urothelium of current and previous smokers [26]. Therefore, in urothelial cells, mutations or other alterations in the p53 pathway caused by cigarette smoking could be involved in the high MNC frequencies observed in our study as well as the increased risk of bladder cancer development and recurrence [12], [13], [19], [20].

Few studies have addressed the aneugenic or clastogenic effects of cigarette smoking in target and surrogate cells. The available data have shown a trend towards no differences between these 2 mechanisms of MN formation [27]. Despite the relatively small sample size, especially after stratification into subgroups, our data demonstrate that subjects with a history of UCC harbor and accumulate genetically unstable cells in the bladder urothelium that may represent early precursors of new UCC or subclones from previous UCC. Furthermore, we showed that the majority of MN detected in urothelial cells arose from chromosomal breakage. Therefore, micronucleus test may provide an informative auxiliary biomarker to monitor bladder cancer.
